# Letter from Aliquie Matchette, U.S.A.

**Published:** 1864-05

**Authors:** Aliquie C. Matchette

**Affiliations:** Small-Pox Hospital, U.S.A., Memphis


### Army Correspondence.

SMALL-POX HOSPITAL, U.S.A., MEMPHIS,
*April* 14, 1864.

Prof. N. S. DAVIS:

SIR:—I have been on duty in the Small-Pox Hospital, at this place, for several months past, and have noted many facts of interest in reference to the fatal and loathsome disease of small-pox.

The location of the hospitals is, every way, good; they are situated one mile east of Main street. The grounds are extensive and beautifully decorated with ornamental and shade trees, and are finely adapted for a place of recreation for the convalescents. The main building is a large brick, two storys high and a basement—is 60 ft. by 36 ft. A large hall, 12 feet in width, divides the building lengthwise, with a cross hall through the middle. Arranged on either side of the halls are rooms 12 ft. square, 10 ft. high—each ventilated and lighted by two large windows—each warmed by a "fire-place," large and of splendid draught, carrying off much foul effluvia. Each room has five patients. The upper part of the house is arranged as the lower floor—*both* floors accommodating only 60 patients. The basement is used as a kitchen, convalescent dining-room, and for store-rooms. The remainder of our patients, over 300, are in common hospital tents—6 to the tent—which is crowding far too closely; yet it has been impossible to do better, as the disease has increased so rapidly as to prevent any better accommodations being provided. Insufficiency of room and proper ventilation, as well as *extremes* of temperature, have been obstacles to the satisfactory treatment of our cases. As pulmonary diseases have often supervened, erysipelas and pymenia frequently attacked the patient, about the time convalescence was fairly beginning—many times, resulting *fatally*, because of the want of *suitable accommodations.* You will see, by the accompanying table, that but a small proportion of the patients have even been vaccinated; therefore we have had a very large number of extremely bad cases—many of the confluent, malignant variety, proving fatal—often within 72 hours after we received them in hospital.

You will not fail to notice the small number of *varioloid* cases. We have had several hundred *negro* soldiers as *patients*, and, as a usual thing, *they* never have been vaccinated, and have small-pox in its most *aggravated* form, with a large proportion of deaths—being very much greater than among whites, everything being equal.

Total number of patients admitted into "Small-Pox Military Hospital," Memphis, Tenn., from 1st January, 1863, to 1st April, 1864 . . . . . . . . . . . 1,498

No. admitted with small-pox . . . . . . . 1,209
" " " varioloid . . . . . . . 289
" of deaths in that time . . . . . . . 379

*First Quarterly Report, for* 1864, *of cases received into Small-Pox Hospital, U.S.A., Memphis, Tenn.*

| MONTHS. | Whole No. of cases admitted. | Vaccinated once or more, successfully. | Never vaccinated. | Varioloid. | Small-pox. | Deaths. |
|---|---|---|---|---|---|---|
| January | 132 | 41 | 91 | 35 | 97 | 21 |
| February | 196 | 62 | 134 | 61 | 135 | 53 |
| March | 295 | 102 | 193 | 66 | 229 | 98 |
| Whole No. Received | 623 | 205 | 418 | 162 | 461 | 172 |

Our treatment has been on general principles, with an external application to face to prevent pitting. The local remedy most beneficial has with us been—℞, Iodine and Glycerine, equal parts, applied freely every two hours, for 24 hours after the eruption appears, which produces desquamation about a week earlier than if left to nature, and leaves the surface smooth and natural.

ALIQUIE C. MATCHETTE, U.S.A.

